# Identifying the most representative actigraphy variables reflecting standardized hand function assessments for remote monitoring in children with unilateral cerebral palsy

**DOI:** 10.1186/s12887-024-04724-z

**Published:** 2024-04-25

**Authors:** Youngsub Hwang, Jeong-Yi Kwon

**Affiliations:** 1 Department of Health Sciences and Technology, Samsung Advanced Institute for Health Sciences and Technology, Sungkyunkwan University, Seoul, Republic of Korea; 2 Department of Physical and Rehabilitation Medicine, Sungkyunkwan University School of Medicine, Samsung Medical Center, Seoul, Republic of Korea

**Keywords:** Actigraphy, Hand function assessment, Principal component analysis, Unilateral cerebral palsy

## Abstract

**Background:**

Accurate assessment of physical activity and motor function in children with cerebral palsy is crucial for determining the effectiveness of interventions. This study aimed to investigate the correlation between real-world activity monitoring outcomes and in-laboratory standardized hand function assessments in children with unilateral cerebral palsy.

**Methods:**

Actigraphy data were collected over 3 days from children aged 4–12 years with unilateral cerebral palsy before in-laboratory assessments. To tackle the high dimensionality and collinearity of actigraphy variables, we first applied hierarchical clustering using the Pearson correlation coefficient as the distance metric and then performed a principal component analysis (PCA) to reduce the dimensionality of our data.

**Results:**

Both hierarchical clustering and PCAs revealed a consistent pattern in which magnitude ratio variables (ln[affected side magnitude/less-affected side magnitude]) were more strongly associated with standardized assessments of hand function than with activity time and distance domain variables. Hierarchical clustering analysis identified two distinct clusters of actigraphy variables, with the second cluster primarily consisting of magnitude ratio variables that exhibited the strongest correlation with Melbourne Assessment 2, Pediatric Motor Activity Log, Assisting Hand Assessment, and Manual Ability Classification System level. Principal component 2, primarily representing the magnitude ratio domain, was positively associated with a meaningful portion of subcategories of standardized measures, whereas principal component 1, representing the activity time and distance component, showed limited associations.

**Conclusions:**

The magnitude ratio of actigraphy can provide additional objective information that complements in-laboratory hand function assessment outcomes in future studies of children with unilateral cerebral palsy.

**Trial registration in ClinicalTrials.gov:**

NCT04904796 (registered prospectively; date of registration: 23/05/2021).

**Supplementary Information:**

The online version contains supplementary material available at 10.1186/s12887-024-04724-z.

## Background

Wearable activity monitors of the present era, such as accelerometers, are revolutionizing biomedical research by providing the ability to approximate real-world conditions and monitor physical activity continuously and impartially [1]. This type of evaluation provides valuable information about general health and daily functioning that may not be revealed by standardized assessments such as questionnaires or in-laboratory tests [2].

The ability to accurately monitor physical activity in real-world conditions has immense potential for clinical applications, particularly in the field of pediatric rehabilitation [3]. Children with cerebral palsy (CP), a group of permanent movement disorders caused by non-progressive brain damage, often experience difficulty with motor function and physical activity [4, 5]. In this respect, accurate and objective monitoring of their physical activity and motor function is crucial for determining the effectiveness of interventions and making informed decisions about their care and treatment. Moreover, traditional standardized assessments and in-laboratory tests, while valuable, may not always provide a comprehensive picture of a child’s functional abilities in their everyday environment [6]. Assessments conducted in a laboratory typically measure an individual’s maximum functional capacity, whereas actigraphy can offer more insight into the actual level of arm use in real-world situations, leading to a better representation of functional performance [7, 8]. The integration of real-world actigraphy data in evaluations, therefore, allows clinicians to gain a more precise and comprehensive understanding of a child’s physical activity and motor function in daily life. It highlights that while laboratory assessments provide insight into potential capacities, actigraphy complements this by illustrating how these capacities are applied in day-to-day activities, thereby offering a holistic view of motor function in children with CP.

While several studies have investigated the correlation between actigraphy outcomes and gross motor function assessments (such as the Gross Motor Function Measure and the 6-minute walk test in children with CP) and have reported a moderate-to-strong correlation [9–11], there is a notable gap in research regarding the relationship between real-world actigraphy data and hand function evaluation outcomes in a laboratory setting for these children. Highlighting the potential of accelerometry in this area, studies among adult populations have shown significant correlations between the accelerometer-derived variables and upper extremity motor function assessments, including the Fugl-Meyer Assessment, Wolf Motor Function Test, and Action Research Arm Test [12, 13]. Yet, in the specific context of CP, especially in assessing upper extremity function, prior research has predominantly focused on data from controlled evaluations, not fully capturing the everyday functional abilities as revealed by real-world actigraphy data [14]. Understanding the relationship between actigraphy data and hand function assessments in children with CP is crucial for healthcare professionals to design and implement individualized intervention plans that address the unique needs of each child. Moreover, given the importance of hand function in completing daily activities and participating in social and recreational activities for children with unilateral CP (UCP) [15], it is essential to investigate the correlation between actigraphy outcomes and hand function test results to obtain a comprehensive understanding of the physical activity and motor function of the upper extremities in this population.

Considering these points, in this study, we aimed to investigate the correlation between physical activity measurements obtained through actigraphy, in-laboratory standardized assessments of hand function and daily activities, and a standardized questionnaire of hand use in children with UCP. We hypothesized that there would be significant correlations between actigraphy-derived physical activity measurements and in-laboratory standardized assessments of hand function and daily activities in children with UCP.

## Methods

### Study overview

This was an observational study nested within a randomized controlled trial of constraint-induced movement therapy (CIMT) for children with UCP conducted at a hospital in Seoul, Republic of Korea. As part of the CIMT study, the children wore accelerometers on both wrists for 3 consecutive days at baseline and after the first (1 month) and second (2 months) intervention phases. To ensure an accurate representation of their typical activity levels, these three-day periods were specifically chosen to be outside the intervention sessions, ensuring that the children were not undergoing intervention during these actigraphy data collection phases. The children underwent standardized assessments, including the Pediatric Motor Activity Log (PMAL), Melbourne Assessment 2 (MA2), AHA, and Pediatric Evaluation of Disability Inventory Computer Adaptive Test (PEDI-CAT). Actigraphy outcomes were analyzed in conjunction with these standardized assessments to investigate their association. The study was approved by the hospital review board (SMC 2021-04-042), and informed consent was obtained from the parents of the children before enrollment.

### Participants

Children aged between 4 and 12 years who were diagnosed with UCP due to central nervous system lesions were recruited to participate in the CIMT study from July 2021 to December 2022. The diagnosis of UCP was made based on the criteria defined by the Surveillance of Cerebral Palsy in Europe, which categorizes UCP based on specific clinical signs and neuroimaging features [16]. Participants were excluded if they had severe cognitive dysfunction that prevented them from performing simple tasks, untreated seizures, visual or auditory problems that could interfere with treatment, or a history of musculoskeletal disorders.

### Physical activity monitoring using accelerometer-based monitors

Physical activity data were collected using ActiGraph wGT3X-BT, and acceleration data were downloaded and converted into 10-s epochs using ActiLife 6 software (ActiGraph, Pensacola, FL, USA) and subsequently converted into activity counts. The accelerometer was programmed to collect data continuously for 3 consecutive days in the week preceding each in-laboratory visit for standardized assessments, and the children were instructed to wear accelerometers on both wrists for at least 12 h per day during the prior 3 days beginning on Friday.

### Selection of actigraphy variables

Acceleration data were recorded in raw format and downloaded using the ActiLife 6 software (ActiGraph). Subsequently, the software generated 52 variables in the time, energy, and distance domains. Considering the study’s small sample size, we opted to reduce the number of actigraphy variables to enhance the stability of the analysis. The selection process involved a comprehensive literature review to identify the most relevant and representative variables for measuring physical activity in our target group population, children with UCP. This review entailed a systematic search of databases for studies utilizing actigraphy in this specific patient population, focusing on identifying variables that were consistently used and shown to be significant in these studies. Key criteria for variable selection included frequency of use in previous research, applicability to our target group population, and the ability to provide meaningful insight into physical activity patterns. The selected variables and references are detailed in Additional File 2. Furthermore, to ensure our selection was well-grounded, we also sought input from experienced occupational therapists familiar with the device and its usage in research and clinical practice.

### In-laboratory standardized assessments

#### Pediatric motor activity log (PMAL)

PMAL evaluates the utilization of a child’s more affected upper limbs during daily tasks. It consists of 22 arm-hand functional tasks, with the collected data being systematically organized. The assessment includes two parts: (1) how often (PMAL HO) and (2) how well (PMAL HW) [17].

### Pediatric evaluation of disability inventory computer adaptive test (PEDI-CAT)

PEDI-CAT is a standardized tool that assesses functional abilities in children with various health conditions. It features a 276-item computer adaptive questionnaire based on caregiver reports and covers four domains, including mobility, daily activities, social/cognitive function, and responsibility [18].

#### Melbourne assessment 2 (MA2)

MA2 is a unilateral upper limb function test that measures unimanual capacity. This criterion-based assessment evaluates four aspects of upper limb movement quality: range of motion (ROM), precision, dexterity, and fluency. The test comprises 14 unimanual tasks that are recorded on video for subsequent scoring [19].

### Assisting hand assessment (AHA)

AHA is a standardized instrument designed for children with UCP. It gauges a child’s ability to use the affected hand in assisting the unaffected hand during various bimanual activities. Assessors review video recordings to score the child’s displayed capacity to use their weaker arm and hand across 22 items [20, 21].

### Statistical analyses

In our statistical analyses, we first sought to understand the relationship between actigraphy variables and standardized functional assessments (AHA, PEDI-CAT, PMAL, and MA2) outcomes, as well as age and sex. We used hierarchical clustering, with the Pearson correlation coefficient serving as the distance metric, to investigate these relationships [22]. Specifically, a “distance” between two variables was defined as one minus the absolute value of their Pearson correlation coefficient (1 - |r|). Thus, if two variables had a correlation coefficient near 1 or -1 (indicating a strong positive or negative relationship), their “distance” would be near 0, suggesting that they belong to the same cluster. Conversely, if two variables had a correlation coefficient near 0 (indicating no relationship), their “distance” would be near 1, reflecting that these variables are not closely related and are less likely to be in the same cluster. Next, to address the high dimensionality and collinearity of the actigraphy variables, we applied a principal component analysis (PCA) to our dataset, selecting the top five principal components (PCs) based on the proportion of variance they explained [23]. Upon reducing dimensionality through principal component analysis, we recognized the necessity of a statistical methodology to appropriately account for the repeated measures inherent in our dataset. Consequently, we implemented a mixed-effect model to address this concern. For variables where significant regression coefficients were not computed, precluding the calculation of confidence intervals in the mixed-effect model, we proceeded with a two-dimensional linear regression to further investigate those relationships. All tests were two tailed, and a p-value of 0.05 was used as the cut-off for statistical significance. All statistical analyses were conducted using SAS 9.4 (SAS Institute Inc., Cary, NC, USA) and R 3.5.0 (R Foundation for Statistical Computing, Vienna, Austria).

## Results

**Fig. 1:**
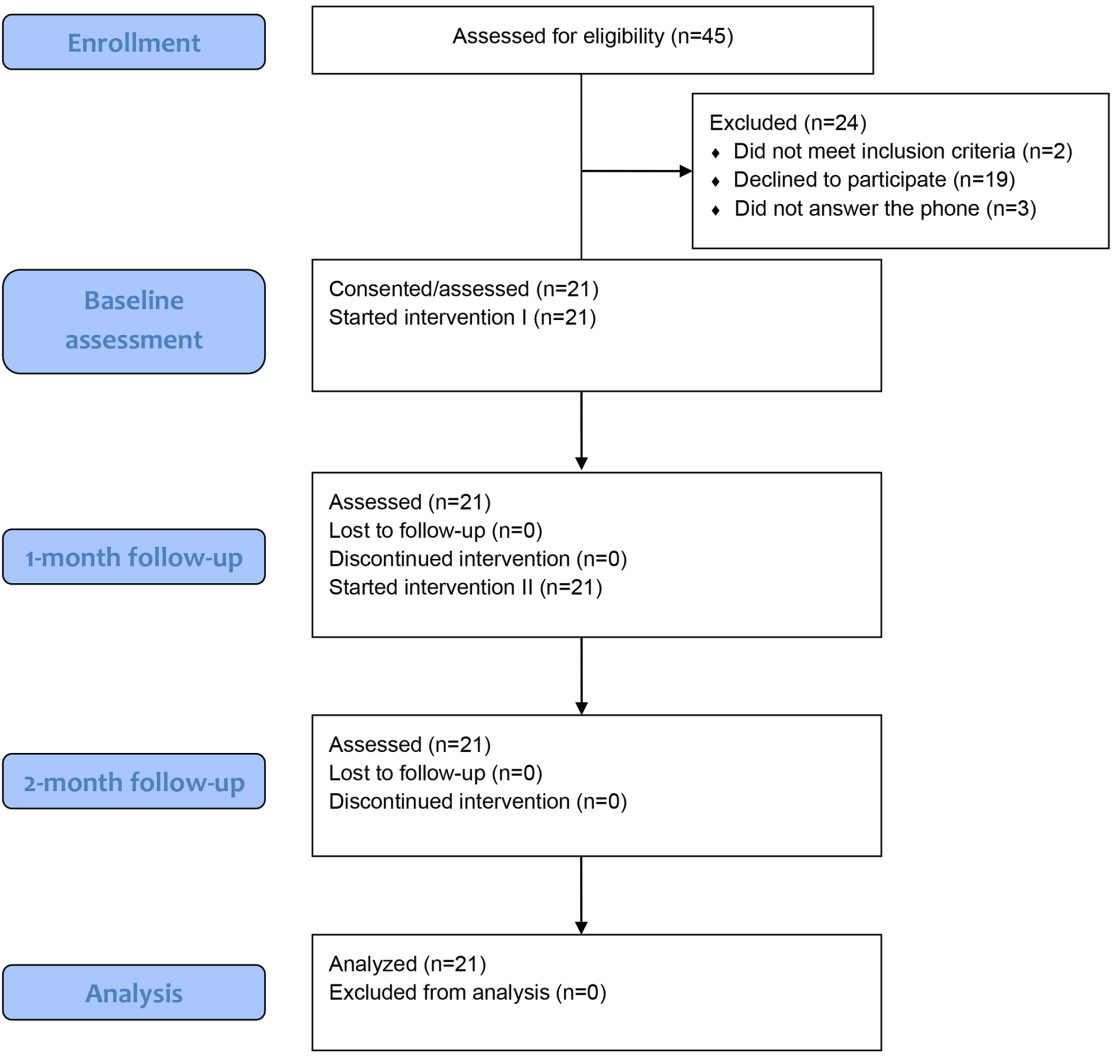
CONSORT 2010 Flow Diagram. CONSORT 2010 flow diagram depicting the progression of participants through the study. Initially, 45 individuals were assessed for eligibility. However, 24 were subsequently excluded for various reasons, as indicated in the diagram. The remaining 21 participants consented to and initiated the first intervention. Follow-up assessments were consistently performed with no loss to follow-up or discontinuation of the intervention at the 1- and 2-month check-ups. Therefore, with each of the 21 participants undergoing three separate assessments, a total of 63 evaluations (*n* = 63) were conducted for the final analysis

A total of 21 children with UCP (12 boys; mean age, 5.56 ± 1.87 years) were included in this analysis (Additional file 1). They wore the actigraphy monitor for 3 consecutive days for at least 12 h per day on three occasions (once a month), yielding a total of 63 data points (Fig. 1). The characteristics of the children are presented in Table 1.

**Table 1:** Demographic and Clinical Characteristics of Children

Characteristics		
**Age, mean (SD), years**		5.56 (1.87)
**Sex, n (%)**	Boys	12 (57.14)
	Girls	9 (42.86)
**MACS level n (%)**	1	7 (33.33)
	2	9 (42.86)
	3	5 (23.81)
**Side of involvement, n (%)**	Right	16 (76.19)
	Left	5 (23.81)

MACS, Manual Ability Classification System; SD, standard deviation

### Selecting suitable actigraphy variables and examining their correlations

The comprehensive review, coupled with expert consultation, led to the selection of eight key variables, further subdivided into criteria such as affected side/less-affected side and axis, resulting in a total of 23 variables (Additional file 2). These variables were categorized based on their primary characteristics and the aspect of physical activity they represent. The selected variables and their definitions are as follows.

1. Vector magnitude average counts (VMA): The average acceleration counts across all three axes of movement (X, Y, and Z). It provides an overall measure of movement intensity, considering movement in all three directions. VMA was further subdivided into the average value (sum of X, Y, Z axes), Axis 1 VMA (X-axis), Axis 2 VMA (Y-axis), and Axis 3 VMA (Z-axis) for both the affected and less-affected sides. This subdivision resulted in a total of 8 distinct variables under the VMA category.
2. VMA ratio: The natural logarithm of the ratio between the affected and less-affected VMA (ln[affected VMA/less-affected VMA]). This calculation was performed for the overall VMA (sum of X, Y, Z axes), as well as separately for each axis (Axis 1 ratio, Axis 2 ratio, and Axis 3 ratio), amounting to 4 variables in total under the VMA ratio category.
3. The sum of VMA: The sum of affected and less-affected average VMAs (sum of X, Y, Z axes).
4. Percentage in sedentary time: The percentage of time an individual spends engaged in sedentary behaviors that do not increase energy expenditure (EE) above the resting level (1.0–1.5 metabolic equivalents [METs]). Two separate variables were calculated for each side.
5. Sedentary time: Time spent in low-intensity activities that do not significantly increase EE above the resting level (1.0–1.5 METs); time (in min) when counts per minute < 100. Two separate variables were calculated for each side.
6. Percentage in moderate-to-vigorous physical activity (MVPA): The percentage of time during a given period that an individual spends in MVPA. Two separate variables were calculated for each side.
7. Average MVPA/hour: Average time spent in MVPA; total MVPA (in min)/total time worn (in hours). Two separate variables were calculated for each side.
8. Average kilocalories (kcal) per hour: Average kcals spent per hour; total kcal/total time worn (in hours). Two separate variables were calculated for each side.

The correlations among the finally selected actigraphy variables are shown in Additional file 3. The variables related to activity time (% in MVPA and average MVPA/hour) and distance (VMA of all axes) were highly associated with each other. Conversely, the magnitude ratio and energy domain exhibited weak correlations with activity time and distance domain, whereas sedentary domain variables (% in sedentary time and sedentary time) showed negative correlations with activity time and distance domain.

### Hierarchical clustering differentiated two groups of actigraphy domains

The hierarchical clustering analysis classified actigraphy domains into two separate clusters (Additional file 4). Variables of activity time and distance domains, including VMA across all axes of both sides, the sum of VMA on both sides, and % in MVPA and average MVPA/hour of both sides clustered together and were strongly negatively correlated with age (Additional file 5). The second cluster consisted primarily of VMA ratio variables for all axes, which exhibited the strongest correlation with MA2, PMAL, AHA, and Manual Ability Classification System (MACS) outcomes (Additional file 6). This cluster also included sedentary time domain variables and energy-related variables.

### PC analysis of the focused dataset of actigraphy variables

**Fig. 2:**
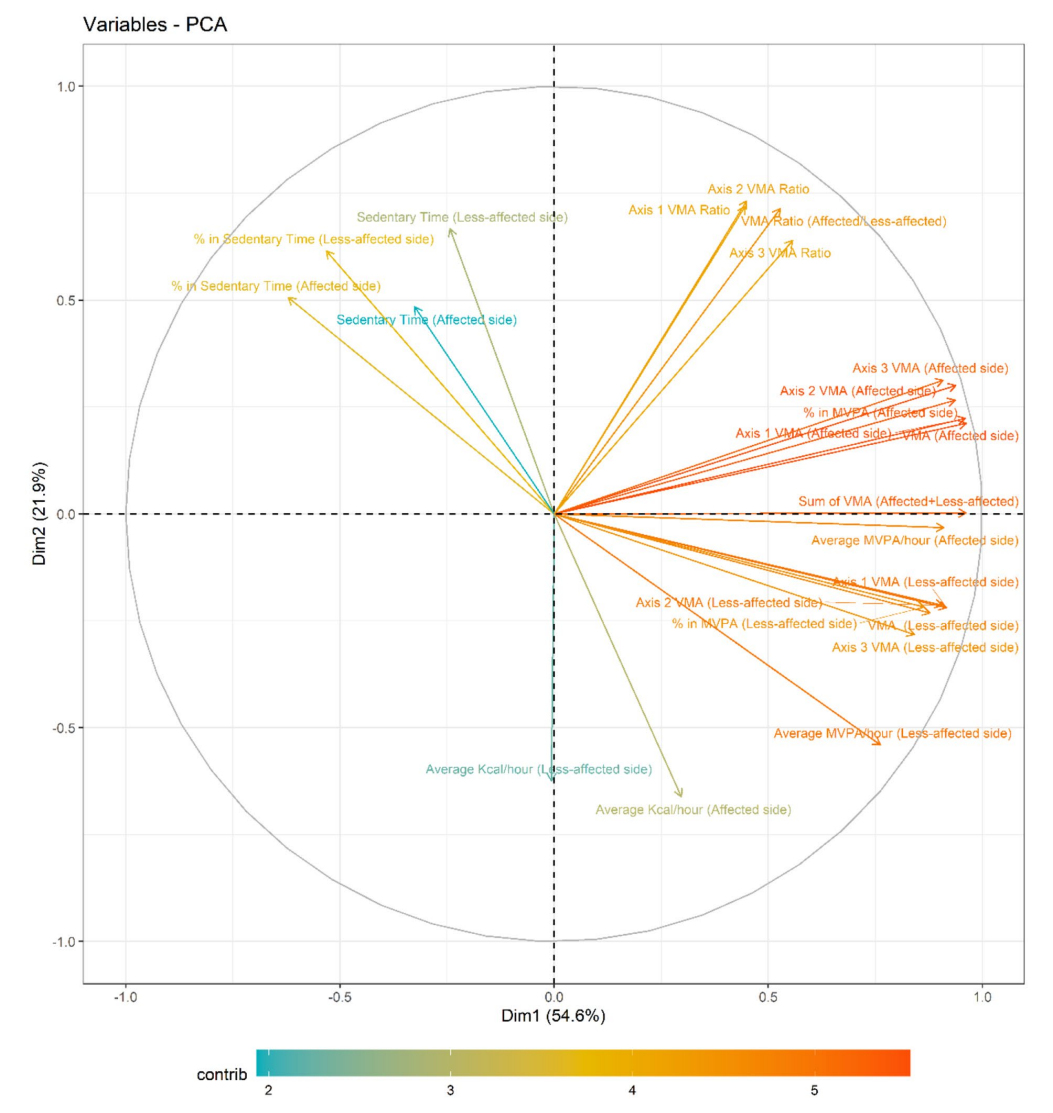
Biplot of Principal Component Analysis (PCA) of the actigraphy variables. The x-axis represents the first principal component (PC1), which explains 54.6% of the total variance and is strongly associated with vector magnitude average counts (VMA) across all axes (both affected and less-affected side), percentage in moderate-to-vigorous physical activity (MVPA; affected side), and average MVPA per hour (affected side). These associations suggest that PC1 primarily represents the activity time and distance measures in actigraphy. The y-axis represents the second principal component (PC2), accounting for 21.9% of the total variance. PC2 is correlated most strongly with the VMA ratio across all axes, sedentary time (less-affected side), and percentage in sedentary time (less-affected side), with an inverse correlation with average kilocalories per hour (both affected and less-affected side). These correlations indicate that PC2 represents the domain of magnitude ratio, sedentary time, and EE in actigraphy. The biplot visually portrays the segregation of these variables along these two PCs.

The first five PCs were selected for analysis, which explained 95.68% of the total variation. Further analysis revealed that the first two components (PC1 and PC2) were the most significant, accounting for 54.6% and 21.9% of the variance, respectively. Together, they represented over 76% of the total variance, enabling a robust interpretation of our data. Given the sharp decrease in explained variance after PC2, including additional components would only increase complexity without significantly improving explanatory power. The most contributing PC (PC1) presented high positive values for the following variables: VMA across all axes (affected side, 0.97; less-affected side, 0.88); VMA of axis 1 (affected side, 0.96; less-affected side, 0.92); VMA of axis 2 (affected side, 0.94; less-affected side, 0.91); VMA of axis 3 (affected side, 0.91; less-affected side, 0.84); the sum of VMA (affected and less-affected side, 0.96); % in MVPA (affected side, 0.94); and average MVPA/hour (affected side, 0.91; Table 2 and Additional file 7). Thus, PC1 was identified as representing activity time and distance measures in actigraphy. The second PC (PC2) exhibited the highest correlation with the VMA ratio of axis 1 (0.72), 2 (0.73), 3 (0.64), and across all axes (0.71). It also displayed positive correlations with sedentary time (less-affected side, 0.67) and % in sedentary time (less-affected side, 0.61) while showing negative correlations with average kcal/hour (affected side, -0.66; less-affected side, -0.62; Table 2 and Additional file 8). PC2 was identified to primarily represent the magnitude ratio domain in actigraphy, with secondary contributions from the sedentary time and EE domains. The biplot in Fig. 2 illustrates the actigraphy variable weights for PC1 and PC2.

**Table 2:** Principal component analysis results

	Principal component				
Actigraphy variables	Dim.1	Dim.2	Dim.3	Dim.4	Dim.5
**VMA (Less-affected side)**	**0.88**	-0.23	0.4	-0.04	0.1
**Axis 1 VMA (Less-affected side)**	**0.92**	-0.22	0.28	0.01	0.05
**Axis 2 VMA (Less-affected side)**	**0.91**	-0.21	0.27	0.02	0.15
**Axis 3 VMA (Less-affected side)**	**0.84**	-0.28	0.37	0.05	0.17
**VMA (Affected side)**	**0.96**	0.21	-0.04	0.04	0.08
**Axis 1 VMA (Affected side)**	**0.96**	0.22	0.06	-0.06	0.03
**Axis 2 VMA (Affected side)**	**0.94**	0.3	0.03	-0.07	0.02
**Axis 3 VMA (Affected side)**	**0.91**	0.31	-0.01	-0.07	0.09
**VMA ratio (Affected/Less-affected)**	0.53	**0.71**	-0.45	0.04	0.01
**Axis 1 VMA ratio**	0.45	**0.72**	-0.47	0.09	0.05
**Axis 2 VMA ratio**	0.45	**0.73**	-0.45	0.04	-0.08
**Axis 3 VMA ratio**	0.56	**0.64**	-0.44	0	0.03
**Sum of VMA (Affected + Less-affected)**	**0.96**	0	0.24	-0.06	0.09
**% in MVPA (Less-affected side)**	**0.86**	-0.22	0.4	-0.08	-0.07
**% in MVPA (Affected side)**	**0.94**	0.27	0.09	-0.11	-0.02
**Average Kcal/hour (Less-affected side)**	-0.01	**-0.62**	-0.62	0.11	0.4
**% in sedentary time (Less-affected side)**	-0.53	**0.61**	0.29	-0.38	0.17
**Sedentary time (Less-affected side)**	-0.24	**0.67**	0.46	0.51	0.04
**Average MVPA/hour (Less-affected side)**	0.76	-0.54	0.02	0.08	-0.31
**Average Kcal/hour (Affected side)**	0.3	**-0.66**	-0.51	0.18	0.18
**% in sedentary time (Affected side)**	-0.62	0.51	0.34	-0.41	0.14
**Sedentary time (Affected side)**	-0.33	0.48	0.54	0.58	0.07
**Average MVPA/hour (Affected side)**	**0.91**	-0.03	-0.2	0	-0.27

VMA, vector magnitude average counts; MVPA, moderate-to-vigorous physical activity

In our PC regression analyses, as presented in Table 3, PC1, encompassing activity time and distance components, did not demonstrate significant associations with in-laboratory assessment outcomes, but revealed a notable correlation with age (*p* < 0.001). In contrast, PC2 primarily influenced by the magnitude ratio, showed significant associations with the PMAL HW (*p* = 0.03) and AHA (*p* = 0.05), alongside correlations with age (*p* < 0.001) and MACS level (*p* < 0.001). This suggests that the magnitude ratio within PC2 is more closely aligned with in-laboratory hand function tests in children with UCP.

**Table 3:** **Linear mixed-effect model analysis incorporating principal components**

Outcomes	PC1 (activity time and distance components)			PC2 (magnitude ratio, sedentary, and energy components)			Model error	Power
	Estimate	95% CI	p	Estimate	95% CI	p	RMSE	
**PMAL**								
How often	0.03	-0.13–0.19	0.75	0.10	-0.05–0.24	0.23	0.34	0.860
How well	0.04	-0.12–0.20	0.62	0.16	0.01–0.30	0.03*	0.20	0.903
**PEDI-CAT**								
Daily activities	-0.01	-0.53–0.52	0.99	0.42	-0.12–0.92	0.08	0.58	0.581
Mobility	0.33	-0.20–0.87	0.24	0.12	-0.38–0.62	0.63	0.60	0.282
Social/Cognitive	-0.07	-0.47–0.33	0.75	-0.12	-0.45–0.22	0.50	0.31	0.610
Responsibility	0.10	-0.63–0.83	0.78	-0.18	-0.76–0.38	0.39	0.60	0.454
**MA2**								
ROM	-0.31	-2.44–1.90	0.77	1.01	-0.69–2.76	0.26	2.33	0.381
Accuracy^**a**^	-0.71	-4.93–2.97	0.70	1.35	-4.46–7.17	0.64	50.52	0.078
Dexterity	-1.12	-4.15–1.88	0.49	1.24	-1.31–3.79	0.35	2.90	0.640
Fluency	-0.44	-1.86–0.98	0.22	0.30	-0.88–1.48	0.64	1.16	0.394
**AHA**	0.19	-0.70–1.10	0.28	0.77	0.03–1.52	0.05*	0.94	0.773
**MACS level** ^**a**^	-0.03	-0.08–0.02	0.25	-0.15	-0.23–-0.07	< 0.001**	0.66	0.962
**Age** ^**a**^	-0.32	-0.41–-0.23	< 0.001**	-0.24	-0.38–-0.09	< 0.001**	1.26	1.000
**Sex, male** ^**a**^	0.04	-0.11–0.18	0.62	-0.07	-0.30–0.15	0.51	91.38	0.050

*, ** Statistically significant values; PMAL, Pediatric Motor Activity Log; PEDI-CAT, Pediatric Evaluation of Disability Inventory Computer Adaptive Test; MA2, Melbourne Assessment 2; AHA, Assisting Hand Assessment; MACS, Manual Ability Classification System; ROM, range of motion; CI, confidence interval; RMSE, root mean square error

^a^Two-dimensional linear regression

## Discussion

This observational study used two analytical strategies to explore the correlation between actigraphy variables and in-laboratory standardized assessments of hand function and daily activities in children with UCP. Notably, the magnitude ratio (ln[affected side VMA/less-affected side VMA]) was strongly correlated with some in-laboratory hand function test variables, which is a key observation in this study. Conversely, the activity time and distance domains did not exhibit such correlations. This discrepancy could arise from the fact that hand function tests target fine motor skills, which require precise movement control but less overall physical activity compared to gross motor function tests, which assess larger movements with less precise control.

The magnitude ratio, a novel biomarker representing the difference in movement quantity between the affected and less-affected upper extremities, has been utilized in previous intervention studies involving children with UCP. Goodwin et al. [24] found that the magnitude ratio could effectively distinguish general hand movement in children with UCP during therapy sessions and outdoor therapy. Additionally, Coker-Bolt et al. [25] reported that a portion of participants (five of twelve) showed improvements in the magnitude ratio following CIMT; our previous study [26] also indicated that a subset of participants (six out of eight) exhibited improvements in the magnitude ratio after CIMT. Our findings highlight the importance of using the magnitude ratio variable within PC2 for future research focused on the upper extremities of children with UCP as it captures a meaningful portion of standardized hand function test outcomes. These insights may have important implications for clinical practice, informing the design and evaluation of targeted interventions aimed at improving hand function and overall quality of life in children with UCP. By focusing on the magnitude ratio and utilizing actigraphy on both wrists, clinicians and researchers can better assess the real-world impact of therapeutic interventions on this population.

Our study also identified an intriguing finding that the activity time and distance components cluster and the magnitude ratio variables cluster demonstrated a strong negative correlation with age in both unsupervised cluster analysis and PC regression approaches. This finding suggests that in children with UCP under 12 years old, movement in the affected upper extremity declines more significantly with age than the less-affected side, even as overall physical activity decreases with age. This result may be because of the progression of developmental disregard with aging. Developmental disregard refers to the behavioral patterns observed in children with UCP who have learned to suppress the use of their more affected upper limb, ultimately leading to neglecting or disregarding it [27]. Our study’s findings suggest a potential decline, which could be interpreted within the context of developmental disregard. However, they do not conclusively establish it as the sole explanation. Future research should aim to explore these nuances further, considering the interplay of biological, psychological, and social factors that contribute to motor development and daily living strategies in children with UCP.

Although no direct evidence indicated a clear correlation between aging and learned non-use in children with UCP, the importance of early interventions in preventing or minimizing developmental disregard is widely recognized [28, 29], implying a potential link between aging and the progression of developmental disregard. However, it is important to note that typically developing children also tend to develop a preference for using one limb as they age. Therefore, future studies that include typically developing children are needed to confirm whether the results are specifically due to learned non-use in children with CP. If actigraphy is demonstrated to be effective in identifying the progression of learned non-use, it could emphasize the importance of using wearable activity monitors in research studies, such as those evaluating the effectiveness of CIMT, as well as other interventions aimed at reducing learned non-use in children with UCP.

The use of activity monitors has seen a significant increase in various medical fields over recent years as they provide objective and reliable data for assessing health status and disease activity by measuring real-world physical activity levels [30]. Wearable devices have become increasingly relevant, particularly because of their use in telehealth as an alternative to in-person care for patients who are unable to access traditional in-center services during the COVID-19 pandemic [30]. Our study demonstrated a strong association between the magnitude ratio variables and the AHA, PMAL, and MA-2, suggesting that changes in standardized assessments after interventions are reflected in this actigraphy variable. This finding emphasizes the importance of carefully selecting and processing actigraphy variables when using this device in telerehabilitation, such as CIMT or bimanual training, to effectively monitor functional hand use levels. The insights from this study can help optimize and refine telerehabilitation plans utilizing actigraphy, potentially contributing to the development of more effective telerehabilitation programs for children with UCP in the future.

### Study limitations

Our study encountered several limitations. First, the short intervals between data collection points could have obscured significant changes in our variables of interest, potentially introducing measurement errors. To address the concern raised regarding the use of 63 data points from 21 children (equivalent to 3 data points per individual) and the adequacy of accounting for repeated measures, we implemented a mixed-effect model analysis. This approach allowed us to interpret the data more accurately, considering the repeated measures inherent in our study design. Considering the small sample size, which could limit the statistical power and our ability to discern meaningful associations, we employed multiple statistical analyses to mitigate these concerns. This study was executed at a single center specializing in children with UCP, hence potentially narrowing the applicability of our results to broader populations or different contexts. Therefore, subsequent research across diverse centers is crucial for validating these findings and ensuring their wider relevance.

## Conclusion

Our research indicates that the magnitude ratio, a key component of actigraphy outcomes within PC2, exhibits a noteworthy correlation with certain standardized measures. This correlation is notably stronger compared to the associations found with the activity time and distance components in PC1. This finding indicates that the magnitude ratio component of actigraphy can provide additional objective information that complements in-laboratory assessment outcomes in future studies of children with UCP. This information can help healthcare professionals determine the effectiveness of interventions and make informed decisions about the care and treatment of children with UCP. Additionally, the study highlights the importance of selecting and processing variables carefully according to the research purpose when using actigraphy. It would be valuable to investigate the relationship between actigraphy outcomes from wrist-worn devices and other standardized assessments, such as gross motor function assessments, to further validate the use of actigraphy in clinical settings.

### Electronic supplementary material

Below is the link to the electronic supplementary material.

Supplementary Material 1

## Data Availability

The data supporting this study’s findings can be obtained from the corresponding author on reasonable request and upon ethical approval.

## Abbreviations

AHA: Assisting Hand Assessment
CIMT: Constraint-induced movement therapy
CP: Cerebral palsy
EE: Energy expenditure
MACS: Manual Ability Classification System
MVPA: Moderate-to-vigorous physical activity
PC: Principal components
PCA: Principal component analysis
PMAL: Pediatric Motor Activity Log
ROM: Range of motion
SCPE: Surveillance of Cerebral Palsy in Europe
VMA: Vector magnitude average counts

## References

[1] Bunn AJ, Navalta WJ, Fountaine JC, Reece DJ (2018). Current state of commercial wearable technology in physical activity monitoring 2015–2017. Int J Exerc Sci.

[2] Prince AS, Adamo BK, Hamel EM, Hardt J, Gorber CS, Tremblay M (2008). A comparison of direct versus self-report measures for assessing physical activity in adults: a systematic review. Int J Behav Nutr Phys Act.

[3] Frontera WR, Bean FJ, Damiano D, Ehrlich-Jones L, Fried-Oken M, Jette A (2017). Rehabilitation research at the National Institutes of Health: moving the field forward (executive summary). Neurorehabil Neural Repair.

[4] Bania TA, Taylor NF, Baker RJ, Graham HK, Karimi L, Dodd KJ (2014). Gross motor function is an important predictor of daily physical activity in young people with bilateral spastic cerebral palsy. Dev Med Child Neurol.

[5] Østensjø S, Carlberg EB, Vøllestad NK (2004). Motor impairments in young children with cerebral palsy: relationship to gross motor function and everyday activities. Dev Med Child Neurol.

[6] Klingels K, De Cock P, Molenaers G, Desloovere K, Huenaerts C, Jaspers E (2010). Upper limb motor and sensory impairments in children with hemiplegic cerebral palsy. Can they be measured reliably?. Disabil Rehabil.

[7] Leanne S, Ziviani J, Boyd RN (2014). Efficacy of upper limb therapies for unilateral cerebral palsy: a meta-analysis. Pediatrics.

[8] Braito I, Maselli M, Sgandurra G, Inguaggiato E, Beani E, Cecchi F (2018). Assessment of upper limb use in children with typical development and neurodevelopmental disorders by inertial sensors: a systematic review. J Neuroeng Rehabil.

[9] Suk MH, Park IK, Yoo S, Kwon JY (2021). The association between motor capacity and motor performance in school-aged children with cerebral palsy: an observational study. J Exerc Sci Fit.

[10] Keawutan P, Bell KL, Oftedal S, Davies PSW, Ware RS, Boyd RN (2018). Relationship between habitual physical activity, motor capacity, and capability in children with cerebral palsy aged 4–5 years across all functional abilities. Disabil Health J.

[11] Mitchell LE, Ziviani J, Boyd RN (2015). Characteristics associated with physical activity among independently ambulant children and adolescents with unilateral cerebral palsy. Dev Med Child Neuro.

[12] Bhatnagar K, Bever CT, Tian J, Zhan M, Conroy SS (2020). Comparing home upper extremity activity with clinical evaluations of arm function in chronic stroke. Arch Rehabil Res Clin Transl.

[13] Lang CE, Wagner JM, Edwards DF, Dromerick AW (2007). Upper extremity use in people with hemiparesis in the first few weeks after stroke. J Neurol Phys Ther.

[14] Beani E, Maselli M, Sicola E, Perazza S, Cecchi F, Dario P (2019). Actigraph assessment for measuring upper limb activity in unilateral cerebral palsy. J Neuroeng Rehabil.

[15] Sakzewski L, Ziviani J, Boyd R (2010). The relationship between unimanual capacity and bimanual performance in children with congenital hemiplegia. Dev Med Child Neuro.

[16] Cans C (2000). Surveillance of cerebral palsy in Europe: a collaboration of cerebral palsy surveys and registers. Dev Med Child Neuro.

[17] Uswatte G, Taub E, Griffin A, Vogtle L, Rowe J, Barman J (2012). The pediatric motor activity log-revised: assessing real-world arm use in children with cerebral palsy. Rehabil Psychol.

[18] Haley SM, Coster WJ, Dumas HM, Fragala-Pinkham MA, Kramer J, Ni P (2011). Accuracy and precision of the Pediatric evaluation of disability inventory computer-adaptive tests (PEDI‐CAT). Dev Med Child Neuro.

[19] Randall M, Imms C, Carey LM, Pallant JF (2014). Rasch analysis of the Melbourne Assessment of Unilateral Upper Limb function. Dev Med Child Neuro.

[20] Holmefur M, Aarts P, Hoare B, Krumlinde-Sundholm Β (2009). Test-retest and alternate forms reliability of the assisting hand assessment. J Rehabil Med.

[21] Krumlinde-Sundholm L, Holmefur M, Kottorp A, Eliasson AC (2007). The assisting Hand Assessment: current evidence of validity, reliability, and responsiveness to change. Dev Med Child Neuro.

[22] Yim O, Ramdeen KT (2015). Hierarchical cluster analysis: comparison of three linkage measures and application to psychological data. Quant Meth Psych.

[23] Jolliffe IT, Cadima J (2016). Principal component analysis: a review and recent developments. Philos Trans Math Phys Eng Sci.

[24] Goodwin BM, Sabelhaus EK, Pan YC, BjornsonKF, Pham KLD, Walker WO (2020). Accelerometer measurements indicate that arm movements of children with cerebral palsy do not increase after constraint-induced movement therapy (CIMT). Am J Occup Ther.

[25] Coker-Bolt P, Downey RJ, Connolly J, Hoover R, Shelton D, Seo NJ (2017). Exploring the feasibility and use of accelerometers before, during, and after a camp-based CIMT program for children with cerebral palsy. J Pediatr Rehabil Med.

[26] Hwang YS, Kwon JY (2020). Effects of modified constraint-induced movement therapy in real-world arm use in young children with unilateral cerebral palsy: a single-blind randomized trial. Neuropediatrics.

[27] DeLuca SC, Echols K, Ramey SL, Taub E (2003). Pediatric constraint-induced movement therapy for a young child with cerebral palsy: two episodes of care. Phys Ther.

[28] Basu AP (2014). Early intervention after perinatal stroke: opportunities and challenges. Dev Med Child Neuro.

[29] Zielinski IM. Developmental disregard in unilateral cerebral palsy: behavioral & electrophysiological examinations of the underlying mechanisms. (Doctoral dissertation, [Sl]:[Sn]). 2017.

[30] Channa A, Popescu N, Skibinska J, Burget R (2021). The rise of wearable devices during the COVID-19 pandemic: a systematic review. Sensors.

