# Supplementary material for: Identifying the most representative actigraphy variables reflecting standardized hand function assessments for remote monitoring in children with unilateral cerebral palsy
Source: BMC Pediatr. 2024 Apr 25;24:273. doi: 10.1186/s12887-024-04724-z (PMC11044557; doi:10.1186/s12887-024-04724-z)
Supplement: Supplementary file 1 — Supplementary Material 1 [file 12887_2024_4724_MOESM1_ESM.docx]

**Additional File**

**Research Article**

Identifying the Most Representative Actigraphy Variables Reflecting Standardized Hand Function Assessments for Remote Monitoring in Children with Unilateral Cerebral Palsy

Youngsub Hwang^1^ and Jeong-Yi Kwon^2,*^

^1^Phd candidate and research assistant, Department of Health Sciences and Technology, Samsung Advanced Institute for Health Sciences and Technology, Sungkyunkwan University, Seoul, Republic of Korea

^2^Professor, Department of Physical and Rehabilitation Medicine, Sungkyunkwan University School of Medicine, Samsung Medical Center, Seoul, Republic of Korea

^*^Correspondence: Jeong-Yi Kwon

Email: [jeongyi.kwon@samsung.com](mailto:jeongyi.kwon@samsung.com)

| **Additional file 1, Supplemental Table 1. Participant Characteristics and Neuroimaging Findings** | | | | | | | | |
| --- | --- | --- | --- | --- | --- | --- | --- | --- |
| Child | Sex | Corrected age (y) | Birth term | Group allocation | Basic pattern of damage | Affected hemisphere | Cortical lesion | Involvement of central nuclei |
| 1 | M | 5.17 | Full | Experimental | MCA infarction | L | F, P | BG |
| 2 | F | 5.00 | Pre | Control | MCA infarction | L | F, P, T | BG, TH |
| 3 | M | 5.42 | Pre | Experimental | N/A | | | |
| 4 | M | 6.50 | Pre | Control | MCA hemorrhagic infarction | L | F, P | BG, TH |
| 5 | F | 5.00 | Full | Experimental | Dysplastic cortex and brainstem, WMDI | L | F, P, T | TH |
| 6 | F | 4.42 | Pre | Control | HIE | L>R | T, P, O | CR |
| 7 | F | 9.58 | Full | Experimental | MCA hemorrhagic infarction | L | F, P, T | BG, TH |
| 8 | M | 4.33 | Pre | Control | Hemorrhage | R | F, P | TH, BG, PO |
| 9 | M | 4.08 | Full | Control | N/A  No MRI  No MRI | | | |
| 10 | M | 10.58 | Pre | Experimental | N/A  No MRI  No MRI  No MRI | | | |
| 11 | F | 4.00 | Pre | Control | WMDI & hemorrhagic infarction | R | F, P, T | BG, TH |
| 12 | M | 9.17 | Pre | Experimental | IVH | L | P, T | BG, TH |
| 13 | M | 4.00 | Full | Control | MCA infarction | L | F, P | TH |
| 14 | M | 4.17 | Full | Experimental | HIE | L | F, P | BG, TH |
| 15 | F | 4.50 | Full | Control | HIE | L | F, P, T | TH |
| 16 | F | 5.08 | Full | Experimental | HIE | R | F | TH, CR |
| 17 | F | 5.83 | Pre | Control | IVH | L | F, P, O | TH |
| 18 | M | 4.33 | Pre | Experimental | WMDI | R, L | F | - |
| 19 | M | 6.00 | Full | Control | MCA hemorrhagic infarction | L | F | BG, TH |
| 20 | M | 4.00 | Full | Experimental | MCA infarction | R | F, P | BG, TH |
| 21 | F | 5.42 | Pre | Experimental | HIE | R | F, P, T | BG, TH |
| M, male; F, female; R, right; L, left; F, frontal; P, parietal; T, temporal; O, occipital; BG, basal ganglia; WMDI, white matter damage of immaturity; MCA, middle cerebral artery; HIE, hypoxic ischemic encephalopathy; -, no finding, TH, thalamus; CR, corona radiata; PO, pons | | | | | | | | |

**Additional file 2, Supplemental Table 2. Utilizing a Literature-guided Approach for Variable Selection in Actigraphy**

| Variable | Domain | Description of variables | Reference |
| --- | --- | --- | --- |
| VMA | Distance | Total of 8 variables, including the overall VMA, and individual axes measurements (Axis 1 VMA, Axis 2 VMA, Axis 3 VMA) for both affected and less-affected sides. | 1-3,7 |
| Sum of VMA | Distance | A single variable representing the combined total of the VMA for both the affected and less-affected sides. | 1 |
| VMA ratio | Magnitude ratio | Total of 4 variables, each representing the natural logarithm (ln) ratio of the affected side to the less-affected side for the overall VMA, Axis 1 VMA, Axis 2 VMA, and Axis 3 VMA. | 1-3 |
| % in sedentary time | Sedentary time | Two variables representing the percentage of sedentary time for both the affected and less-affected sides. | 4,7 |
| Sedentary time | Sedentary time | Two variables indicating the total time spent in low-intensity activities for both the affected and less-affected sides. | 5-6 |
| % in MVPA | Activity time | Two variables detailing the percentage of time spent in MVPA for both the affected and less-affected sides. | 3,7 |
| Avg. MVPA/hour | Activity time | Two variables calculating the average duration of MVPA per hour of device wear for both the affected and less-affected sides. | 5,9 |
| Avg. kcal/hour | Energy | Two variables measuring energy expenditure (kcal) per hour for both the affected and less-affected sides. | 7-8 |

VMA, vector magnitude average counts; EE, energy expenditure; MVPA, moderate-to-vigorous physical activity


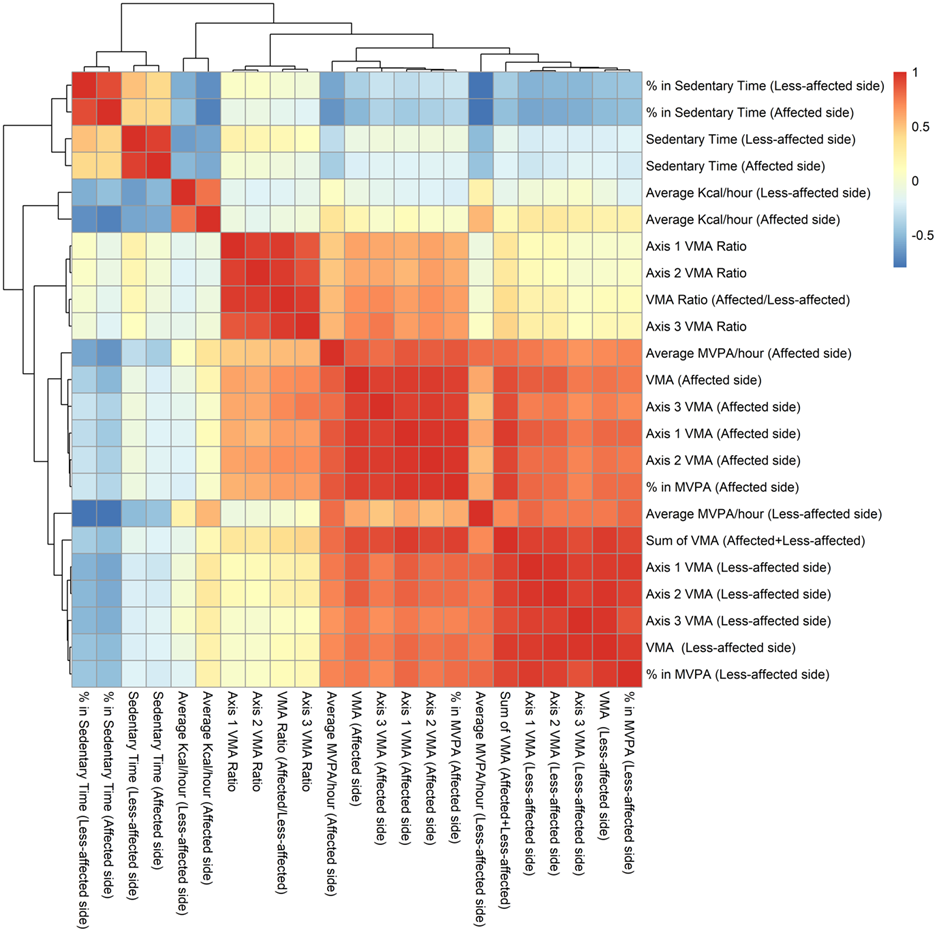


**Additional file 3, Supplemental Figure 1. Correlations Among the Finally Selected Actigraphy Variables.** Using Pearson’s correlation coefficients as the distance metric, we conducted hierarchical clustering, and the resulting dendrograms are presented on top of the heat map to illustrate the clustered relationships among the variables.

MVPA, moderate-to-vigorous physical activity; VMA, vector magnitude of activity

**Additional file 4****, Supplemental Table 3.** **Hierarchical Clustering Analysis Categorized Actigraphy Domains into Two Distinct Clusters**

| Variable | Cluster |
| --- | --- |
| VMA (Less-affected side) | 1 |
| Axis 1 VMA (Less-affected side) | 1 |
| Axis 2 VMA (Less-affected side) | 1 |
| Axis 3 VMA (Less-affected side) | 1 |
| VMA (Affected side) | 1 |
| Axis 1 VMA (Affected side) | 1 |
| Axis 2 VMA (Affected side) | 1 |
| Axis 3 VMA (Affected side) | 1 |
| % in MVPA (Less-affected side) | 1 |
| % in MVPA (Affected side) | 1 |
| Average MVPA/hour (Less-affected side) | 1 |
| Average MVPA/hour (Affected side) | 1 |
| Sum of VMA (Affected + Less-affected side) | 1 |
| VMA Ratio (Affected/Less-affected) | 2 |
| Axis 1 VMA ratio | 2 |
| Axis 2 VMA ratio | 2 |
| Axis 3 VMA ratio | 2 |
| Average kcal/hour (Less-affected side) | 2 |
| % in sedentary time (Less-affected side) | 2 |
| Sedentary time (Less-affected side) | 2 |
| Average kcal/hour (Affected side) | 2 |
| % in sedentary time (Affected side) | 2 |
| Sedentary time (Affected side) | 2 |

VMA, vector magnitude average counts; MVPA, moderate-to-vigorous physical activity


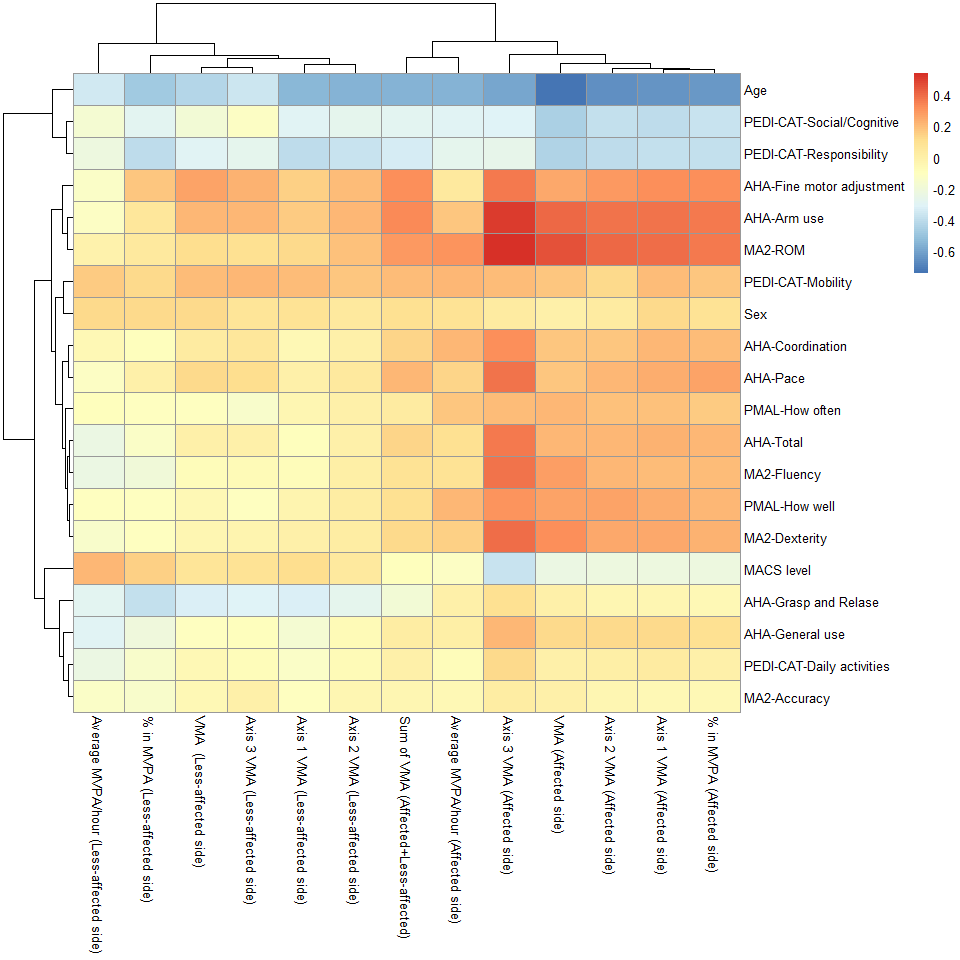


**Additional file 5****, Supplemental Figure 2. Correlations Between Cluster 2 Actigraphy Variables and Standardized Assessments.** Using Pearson’s correlation coefficients as the distance metric, we conducted hierarchical clustering, and the resulting dendrograms are presented on top of the heat map to illustrate the clustered relationships among the variables.

AHA, Assisting Hand Assessment; PEDI-CAT, Pediatric Evaluation of Disability Inventory Computer Adaptive Test; PMAL, 2.5.1 Pediatric Motor Activity Log; MACS, Manual Ability Classification System; MA 2, Melbourne Assessment 2; MVPA, moderate-to-vigorous physical activity; VMA, vector magnitude average counts; ROM, range of movement


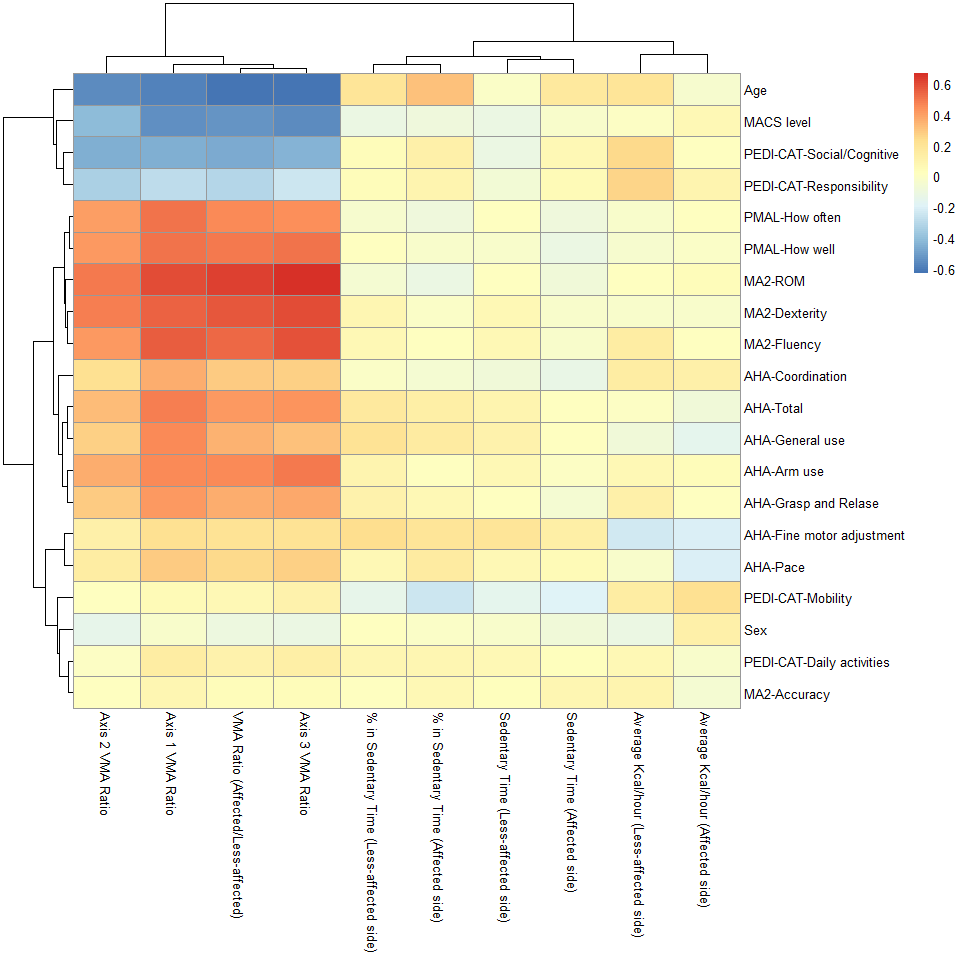


**Additional file 6****, Supplemental Figure 3. Correlations Between Cluster 1 Actigraphy Variables and Standardized Assessments.** Using Pearson’s correlation coefficients as the distance metric, we conducted hierarchical clustering, and the resulting dendrograms are presented on top of the heat map to illustrate the clustered relationships among the variables.

AHA, Assisting Hand Assessment; PEDI-CAT, Pediatric Evaluation of Disability Inventory Computer Adaptive Test; PMAL, 2.5.1 Pediatric Motor Activity Log; MACS, Manual Ability Classification System; MA 2, Melbourne Assessment 2; MVPA, moderate-to-vigorous physical activity; VMA, vector magnitude average counts; ROM, range of movement

**
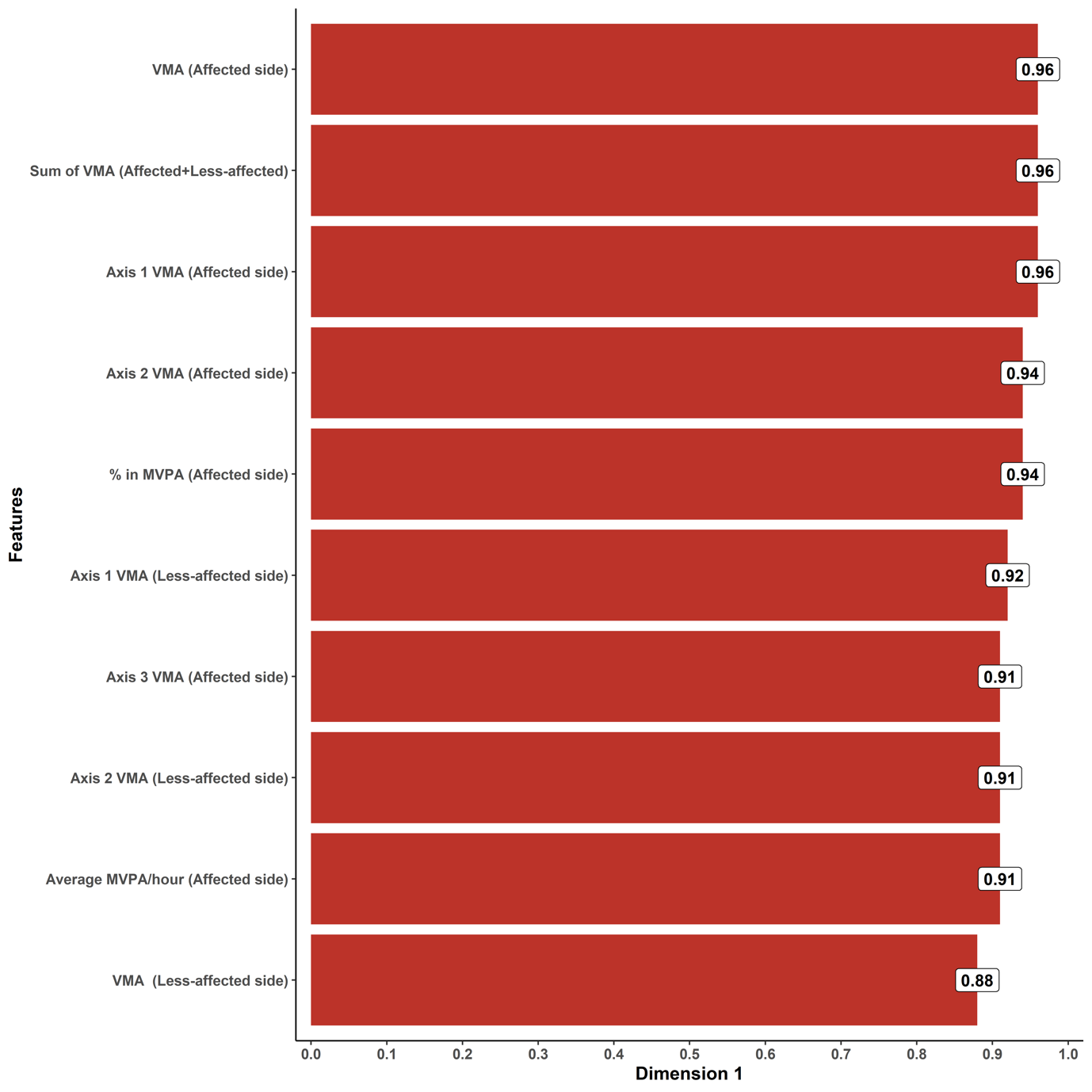
**

**Additional file 7****, Supplemental Figure 4. Components of the Most Contributing PC (PC1).** PC1 shows high positive values for the following parameters: VMA across all axes, VMA of axis 1, VMA of axis 2, VMA of axis 3, sum of VMA, % in MVPA, and average MVPA/hour.

PC, principal component; MVPA, moderate-to-vigorous physical activity; VMA, vector magnitude average counts

**
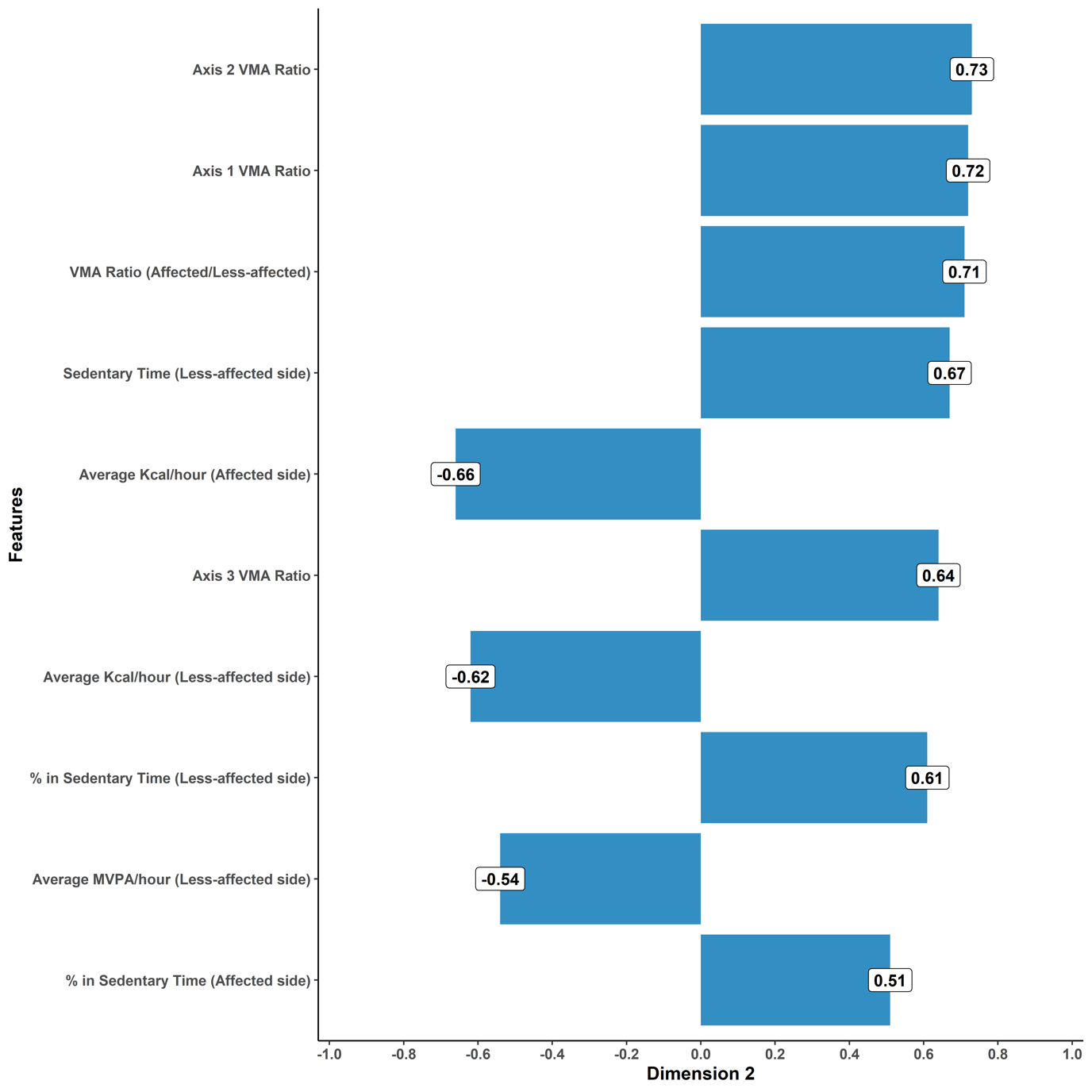
**

**Additional file 8****, Supplemental Figure 5. Components of PC2.** PC2 is highly correlated with the following parameters: VMA ratio across all axes, sedentary time, % in sedentary time, average kcal/hour, and average MVPA/hour.

PC, principal component; MVPA, moderate-to-vigorous physical activity; VMA, vector magnitude average counts

**Additional File References**

1. Goodwin BM, Sabelhaus EK, Pan YC, Bjornson KF, Pham KLD, Walker WO, et al. Accelerometer measurements indicate that arm movements of children with cerebral palsy do not increase after constraint-induced movement therapy (CIMT). Am J Occup Ther. 2020;74:7405205100p1-9.
2. Coker-Bolt P, Downey RJ, Connolly J, Hoover R, Shelton D, Seo NJ. Exploring the feasibility and use of accelerometers before, during, and after a camp-based CIMT program for children with cerebral palsy. J Pediatr Rehabil Med. 2017;10:27-36.
3. Hwang YS, Kwon JY. Effects of modified constraint-induced movement therapy in real-world arm use in young children with unilateral cerebral palsy: a single-blind randomized trial. Neuropediatrics. 2020;51:259-66.
4. Keawutan P, Bell KL, Ofteda S, Ware RS, Stevenson RD, Davies PSW, et al. Longitudinal physical activity and sedentary behaviour in preschool‐aged children with cerebral palsy across all functional levels. Dev Med Child Neurol. 2017;59:852-7.
5. Claridge EA, McPhee PG, Timmons BW, Martin GKA, Macdonald MJ, Gorter JW. Quantification of physical activity and sedentary time in adults with cerebral palsy. Med Sci Sports Exerc. 2015;47:1719-26.
6. Smit DJM, Zwinkels M, Takken T, Hulst RY, de Groot JF, Lankhorst K, Verschuren O. Sleep quantity and its relation with physical activity in children with cerebral palsy; insights using actigraphy. J Paediatr Child Health. 2020;56:1618-22.
7. Lee J, Suk MH, Yoo S, Kwon JY. Physical activity energy expenditure predicts quality of life in ambulatory school-age children with cerebral palsy. J Clin Med. 2022;11:3362.
8. Xing R, Huang WY, Sit CH. Validity of accelerometry for predicting physical activity and sedentary time in ambulatory children and young adults with cerebral palsy. J Exerc Sci Fit. 2021;19:19-24.
9. McPhee PG, Brunton LK, Timmons BW, Bentley T, Gorter JW. Fatigue and its relationship with physical activity, age, and body composition in adults with cerebral palsy. Dev Med Child Neurol. 2017;59:367-73.
